# A Case of Simultaneous Overlapping Cancer of the Pancreatic Head and Gastric Body Managed With Pancreaticoduodenectomy and Total Gastrectomy in Two Stages

**DOI:** 10.7759/cureus.83777

**Published:** 2025-05-09

**Authors:** Mana Kawajiri, Masaki Kimura, Koya Tochii, Toshiya Higashi, Hidetoshi Matsunami

**Affiliations:** 1 Surgery, Matsunami General Hospital, Gifu, JPN

**Keywords:** gastric cancer surgery, pancreatic cancer treatments, pancreaticoduodenectomy (pd), simultaneous overlapping cancer, total gastrectomy

## Abstract

Simultaneous overlapping cancers are defined as two or more primary malignancies diagnosed within six months of each other. The treatment strategy is highly individualized and depends on the localization and progression of each cancer. Although radical surgery is sometimes performed at the first instance, patients might be hesitant to undergo extensive resection due to the risk of surgical complications, depending on their condition. Herein, we describe the case of an 80-year-old male patient, diagnosed with concurrent pancreatic head and upper gastric cancer. He underwent a two-stage surgical treatment with favorable results.

The patient was initially diagnosed with gastric cancer (cT1b, cN0, cM0, cStage1) and pancreatic head cancer (cT1, cN0, cM0, cStage1A) based on gastrointestinal endoscopy and computed tomography findings. Due to the highly invasive nature of simultaneous surgical resection, the patient opted to avoid a one-stage surgical resection. Furthermore, the treatment plan prioritized surgery for pancreatic cancer, which included subtotal stomach-preserving pancreaticoduodenectomy, m-Child reconstruction, and the Blumgart method. Postoperative pathology showed a pT3 (with duodenal invasion), pN0, cM0, and pStage2A pancreatic cancer. The patient received S-1 (tegafur/gimeracil/oteracil potassium) as an adjuvant chemotherapy for pancreatic cancer. This treatment resulted in a significant shrinkage of the gastric cancer after three months. However, one year after the surgery, the gastric cancer progressed, spreading from the upper gastric body to the middle. Despite administering two courses of S-1, it did not shrink. A total gastrectomy with Roux-en-Y reconstruction was performed, and the pathological findings indicated ypT1b, ypN0, ycM0, and ypStage1A gastric cancer. The patient remained alive even 31 months after the surgery, without recurrence.

Our patient was an older adult, raising concerns about the complications with one-stage surgical resection. We successfully managed the patient by adopting a two-stage surgical approach with adjuvant chemotherapy in between. It resulted in recurrence-free survival at 31 months after the operation.

## Introduction

The incidence of multiple primary cancers among patients with pancreatic cancer is reported to be 8.4% [1]. This figure is notably higher in Japan at 13.2% [2], surpassing the rates observed with other malignant tumors. The treatment strategy for concurrent cancers depends on the localization and stage of the disease. Surgery for pancreatic cancer is highly invasive, and following gastric cancer surgery, maintaining proper nutritional status can be challenging due to the effects of gastric resection. Simultaneous surgery for pancreatic and gastric cancers, particularly in older patients or those at high risk for surgical complications due to preexisting disease, might lead to a further decline in activities of daily living and nutritional status after surgery. In this report, we present a case of an older patient diagnosed with both pancreatic head and gastric cancers who underwent a two-stage surgical treatment and achieved favorable outcomes. This article was previously presented as a meeting abstract at the 2023 Japanese Gastric Cancer Association (JGCA) Annual Meeting on February 24, 2023.

## Case presentation

All cancer staging in this report was performed according to the 8th edition of the Union for International Cancer Control Tumour, Node, Metastasis (UICC TNM) classification [3].

**Figure 1:**
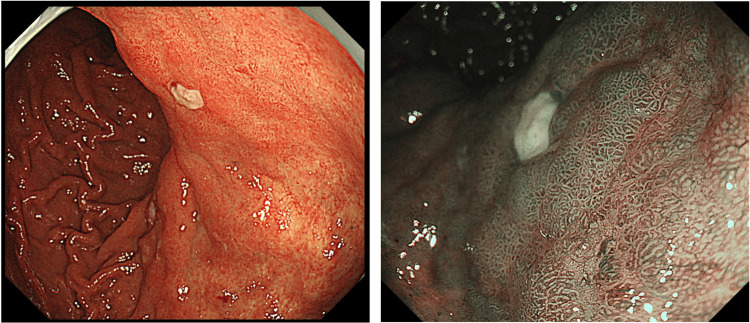
EGD at initial diagnosis Upper gastrointestinal endoscopy revealed a 30mm, Type B1 lesion on the posterior wall of the upper gastric body. The image on the right shows narrow band imaging. EGD: Esophagogastroduodenoscopy

An 80-year-old man presented to our hospital with a chief complaint of upper abdominal pain on an empty stomach that had started a week earlier. Upper gastrointestinal endoscopy revealed a 30 mm, Type B1 lesion on the posterior wall of the upper gastric body (Figure 1).

**Figure 2:**
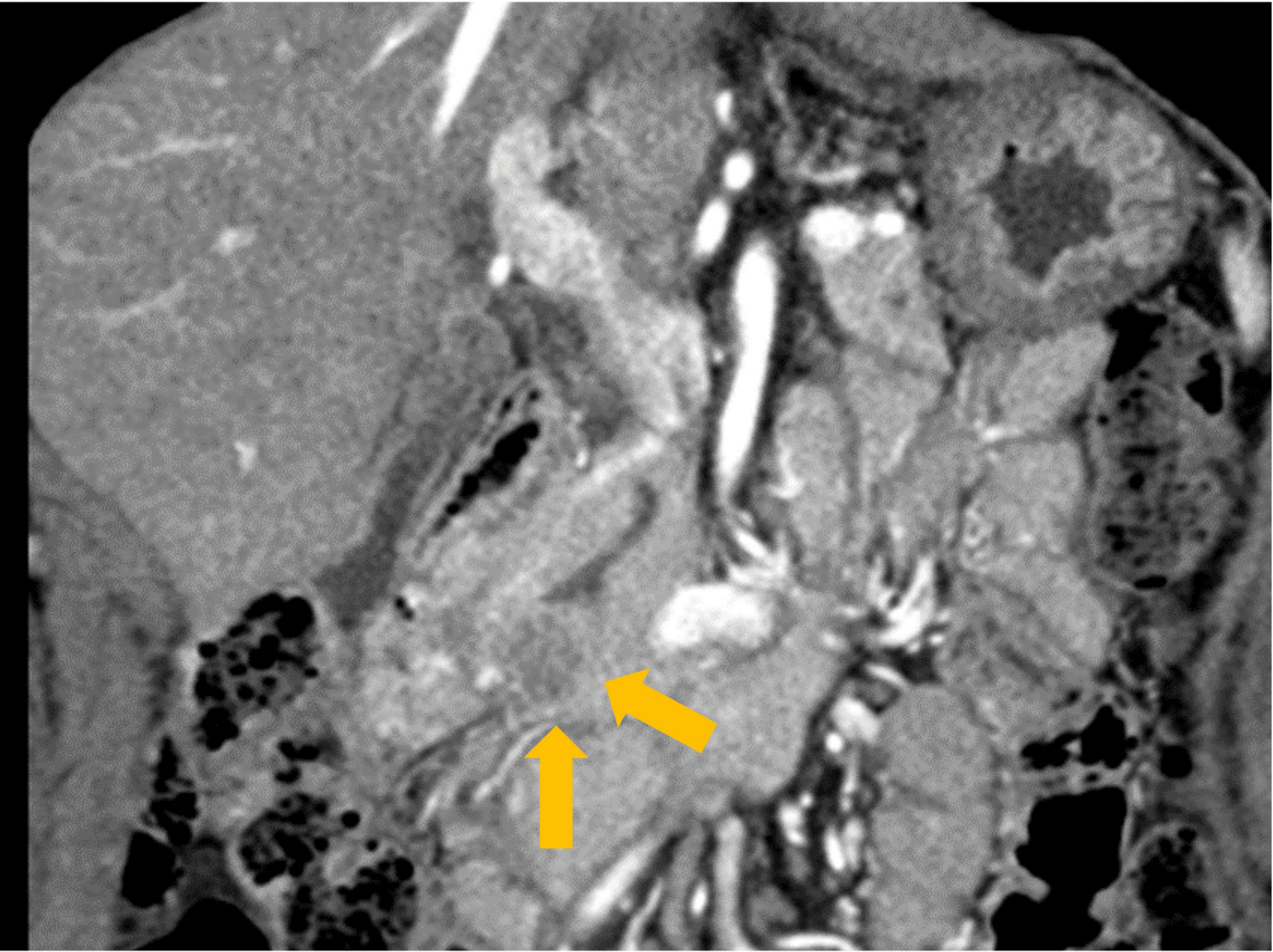
Contrast CT (coronal) There is a 20 mm hypovascular tumor in the pancreatic hook area along with stenosis of the main pancreatic duct at the same site.

Contrast-enhanced computed tomography (CT) showed no obvious lymph node enlargement or distant metastases. A 20 mm hypovascular tumor was identified in the pancreatic hook area, along with stenosis of the main pancreatic duct at the same site (Figure 2).

**Figure 3:**
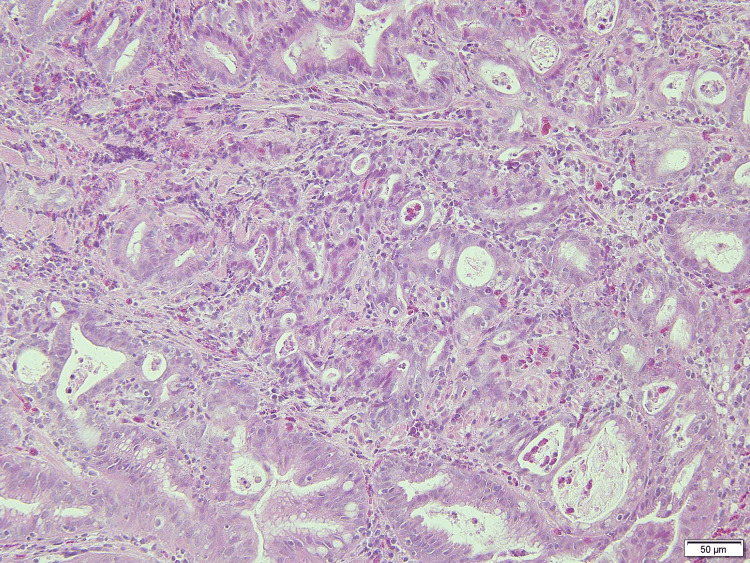
Biopsy specimen of the gastric cancer A medium-differentiated tubular adenocarcinoma is seen.

The tumor markers carcinoembryonic antigen (CEA) and cancer antigen 19-9 (CA19-9) were not elevated and were at 2.0 ng/ml (reference value: <5.0 ng/ml) and 25.1 U/ml (reference value: <37.0 U/ml), respectively. Biopsy and CT scan results confirmed a diagnosis of gastric cancer (cT1b, cN0, cM0, cStage1) (Figure 3) and pancreatic cancer (cT1, cN0, cM0, cStage1A) indicating simultaneous overlapping cancers. 

**Figure 4:**
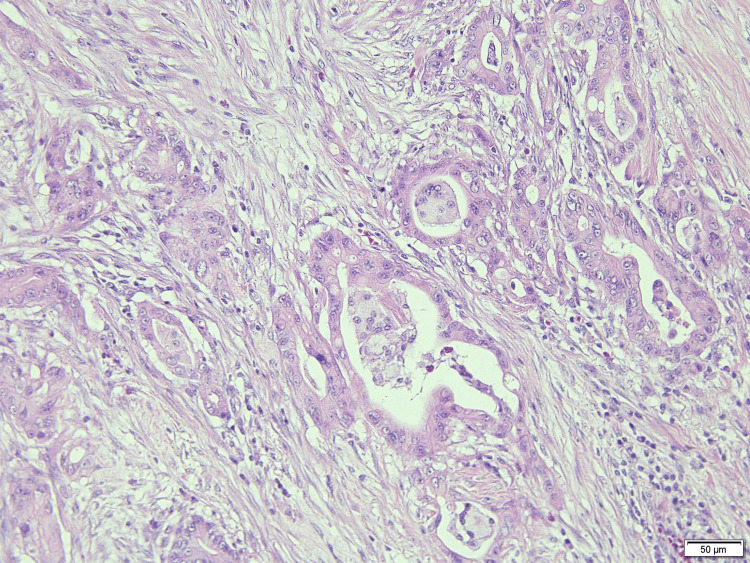
Surgical pathology specimen of pancreatic cancer The postoperative pathological results were pT3 (with duodenal invasion), pN0, cM0, and pStage2A

Considering the patient's age, advanced stage, and surgical invasiveness, as well as the fact that the patient did not want a one-stage resection, the treatment plan involved an initial pancreatic cancer surgery followed by a two-stage treatment of gastric cancer. The patient underwent a subtotal stomach-preserving pancreaticoduodenectomy with reconstruction using the Child’s modification technique. The duration of the operation was 322 min, with a blood loss of 200 ml. The postoperative pathological results were pT3(with duodenal invasion), pN0, cM0, and pStage2A (Figure 4).

**Figure 5:**
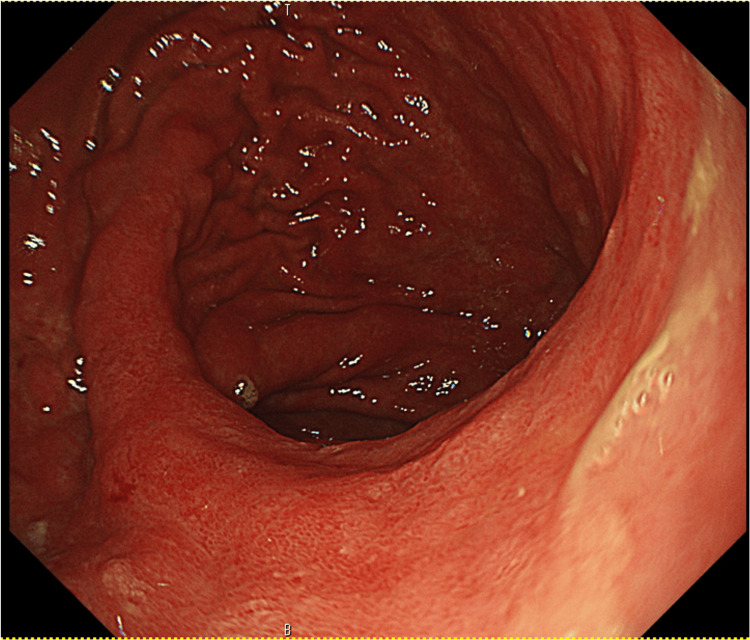
EGD three months after the surgery The gastric cancer has shrunk significantly, and the biopsy did not show any tumor cells. EGD: Esophagogastroduodenoscopy

The patient was administered S-1 (tegafur/gimeracil/oteracil potassium) as adjuvant chemotherapy (80mg/day, four weeks on and two weeks off). Three months later, upper gastrointestinal endoscopy showed significant shrinkage of the gastric tumor, and the biopsy did not show any tumor cells (Figure 5).

**Figure 6:**
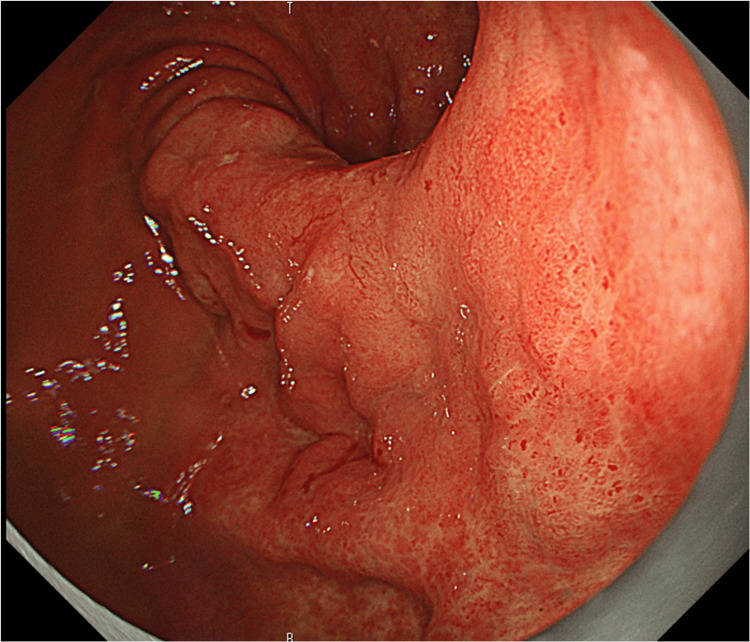
EGD one year after the surgery Coarse mucosal changes were observed in the upper and middle gastric body, and biopsy result showed a moderately differentiated adenocarcinoma. EGD: Esophagogastroduodenoscopy

However, one year after surgery, an upper gastrointestinal endoscopy revealed coarse mucosal changes in the upper to middle gastric region. A biopsy confirmed an intermediate-differentiated adenocarcinoma, indicative of gastric cancer progression (Figure 6).

**Figure 7:**
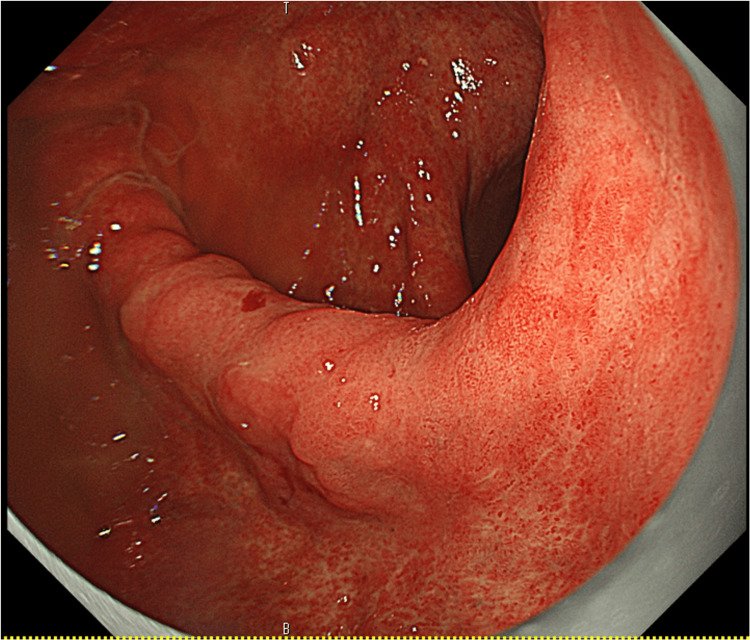
EGD performed after two courses of chemotherapy No reduction in gastric cancer is observed. EGD: Esophagogastroduodenoscopy

As deep submucosal invasion was suspected, endoscopic treatment was not indicated and S-1 treatment was resumed. No reduction in the gastric cancer was observed even after two courses of chemotherapy (Figure 7).

**Figure 8:**
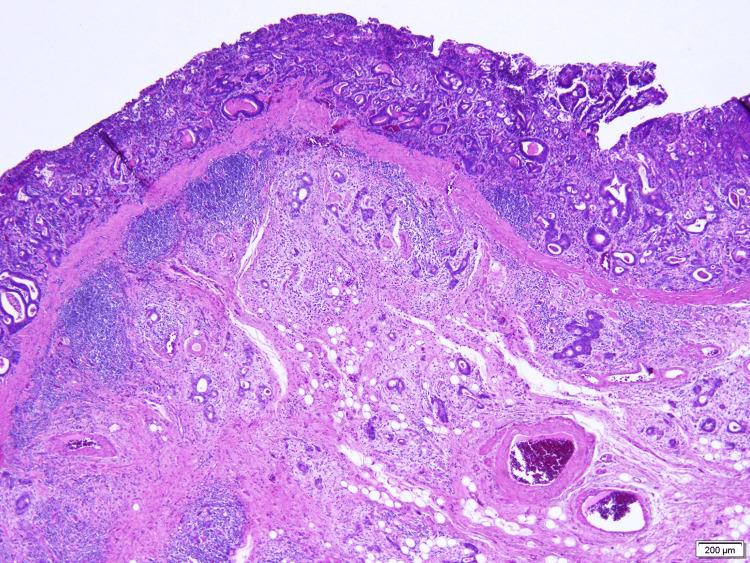
Surgical pathology specimen of the gastric cancer Tumor cells have invaded the submucosa beyond the mucosal muscle plate.

Contrast-enhanced CT showed no evidence of lymph node involvement or distant metastasis and recurrence of the pancreatic cancer was not detected. Surgery was planned and the patient underwent a total abdominal gastrectomy with a D2 dissection (standard lymphadenectomy) and Roux-en-Y reconstruction. The jejunum, which had been previously anastomosed with the pancreas and the bile duct, was re-anastomosed with the ascending jejunum and terminal anastomosis to form a Y-leg. The duration of the operation was 212 min, with blood loss of 10 ml. The final diagnoses were ypT1b, ypN0, ycM0 and yp Stage1A (Figure 8).

**Figure 9:**
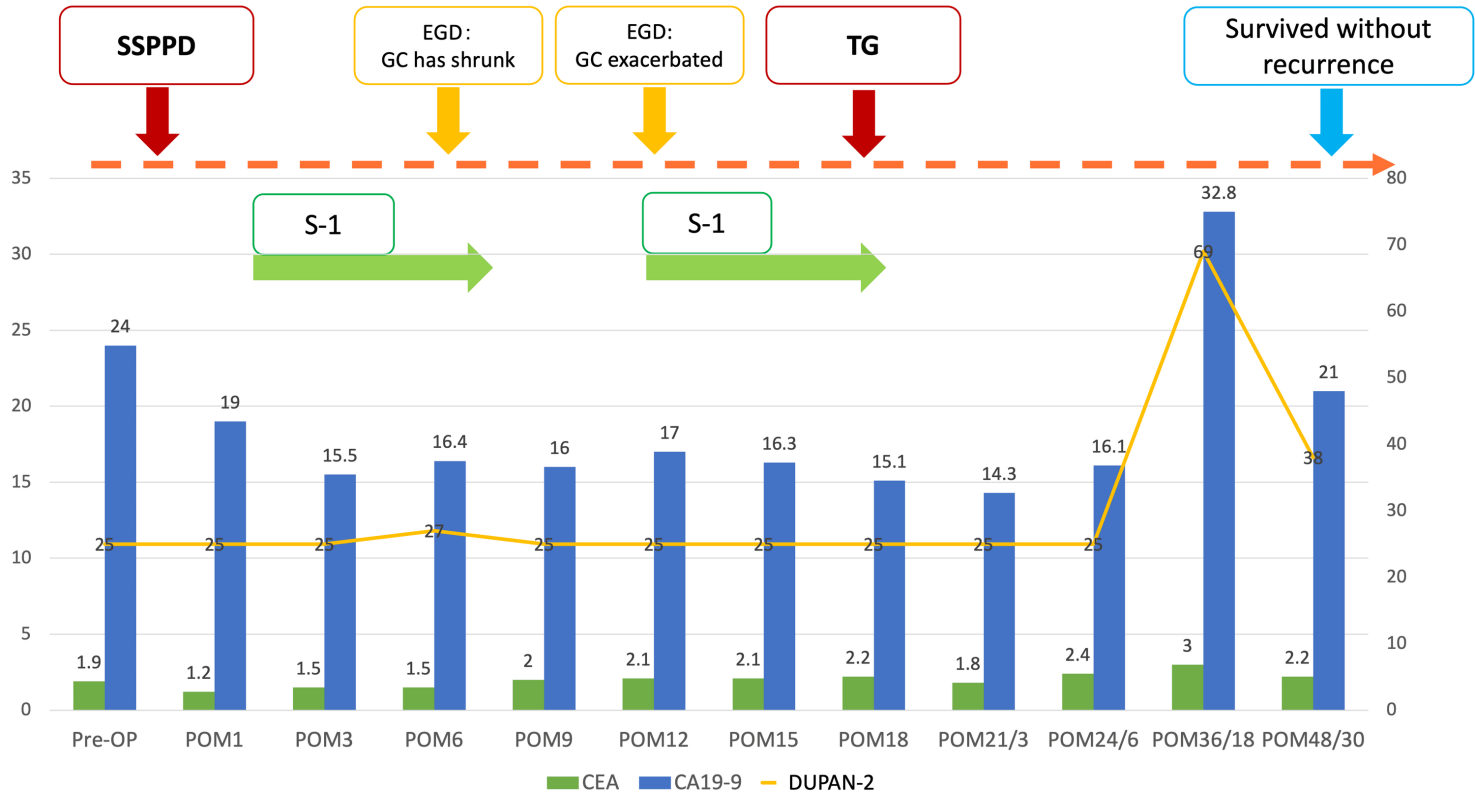
Course of treatment SSPPD: Subtotal stomach-preserving pancreatoduodenectomy; EGD: Esophagogastroduodenoscopy; GC: Gastric cancer; TG: Total gastrectomy; S-1: tegafur/gimeracil/oteracil potassium; Pre-OP: preoperative; POM: postoperative month; CEA: Carcinoembryonic antigen; CA19-9: cancer antigen19-9; DUPAN-2: Duke Pancreatic Monoclonal Antigen Type 2

The patient was discharged on postoperative day 15. At 49 months after the pancreatic cancer surgery and 31 months after the gastric cancer surgery, the patient remained alive with no evidence of disease recurrence (Figure 9).

## Discussion

The frequency of multiple cancers in patients with pancreatic cancer is reported to be 8.4% [1]. The frequency of pancreatic cancer overlapping with cancers of other organs varied among previous reports. However, gastrointestinal and thyroid cancers were relatively frequent, with one study reporting a high overlap rate of 42.3% [4] with stomach cancer in Japan. We searched the Japanese Central Medical Journal and PubMed databases for cases of overlapping pancreatic and gastric cancers. After excluding conference proceedings, in addition to the present case, we identified and reviewed 15 cases of concurrent overlapping cancers of the stomach and pancreas reported between 2000 and 2024 (Tables 1, 2) [5-18].

**Table 1:** Details of the 12 surgical cases of concurrent overlapping gastric and pancreatic cancers [5-15] M: Male; F: Female; PaC: Pancreatic cancer; Ph: Pancreatic head; Pt: Pancreatic tail; PDAC: Pancreatic ductal adenocarcinoma; IPMC: Intraductal papillary mucinous carcinoma; SCC: Squamous cell carcinoma; GC: Gastric cancer; CCH: Cholangiocarcinoma; SC: Sigmoid colon cancer; PD: Pancreaticoduodenectomy; TG: Total gastrectomy; DP: Distal pancreatectomy; GEM: gemcitabine; rC: recurrence; tub1: well-differentiated adenocarcinoma; tub2: moderately-differentiated adenocarcinoma; por: poorly-differentiated adenocarcinoma; por2: poorly-differentiated adenocarcinoma, non-solid type; SOX: S-1 plus oxaliplatin therapy; S-1: tegafur/gimeracil/oteracil potassium; POM: postoperative month; subTG: subtotal gastrectomy

	Age	Sex	Localization of PaC	Organization type	Stage	Localization of GC	Organization type	Stage	Other complicated cancers	Operation	Adjuvant chemotherapy	Postoperative course
Eriguchi, 2000	76	M	Pb	PDAC	1	Middle third	tub	1B	none	DP+subTG	none	POM13 death
Ishiguro, 2001	61	M	Ph	IPMC	1A	Middle third	por2	1B	none	PD+TG	none	POM10 alive
Nakatsuji, 2003	64	M	Ph	SCC	2B	Middle third	por2	1A	none	PD+TG	none	POM2 rC,POM5 death
Mizuno, 2004	87	M	Ph	IPMC	2A	Lower third	tub1	1A	CCH (Stage2)	PD	none	POM11 alive
Yoshida, 2006	66	M	Pt	SCC	2	Lower third	por2	2B	none	DP+TG	none	POM7 rC,POM13 death
Ouchi, 2009	78	F	Ph	PDAC	3	Middle third	por2	2B	SC (Stage3)	PD+Sigmoidectomy	none	POM16 death
Tanaka, 2009	78	M	Ph	SCC	2B	Middle third	tub2	1B	none	PD+subTG	none	POM3 rC,POM5.5 death
Kajiwara, 2012	72	M	Ph	PDAC	3	Middle third	tub1	1A	none	PD+subTG	GEM	POM13 death
Santos, 2015	64	F	Pt	PDAC	4 (Liver)	Lower third	tub1	1	none	DP+subTG+Partial Hepatectomy	not mentioned	not mentioned
Tian, 2021	69	M	Pt	PACC	3	Lower third	por	3B	none	DP+TG	SOX	not mentioned
Ebihara, 2024	59	M	Pb	PDAC	2B	Upper third	por+sig	2A	none	Lap-DP+TG	S-1	not mentioned
Our case	80	M	Ph	PDAC	2A	Middle third	tub2	1A	none	PD→TG	S-1	POM49/POM31 alive

**Table 2:** Details of the four cases of concurrent overlapping gastric and pancreatic cancer treated with chemotherapy [16-18] F: Female; M: Male; PaC: Pancreatic cancer; Ph: Pancreatic head; Pt: Pancreatic tail; PDAC: Pancreatic ductal adenocarcinoma; GC: Gastric cancer; PALN: Para-aortic lymph nodes; por: poorly-differentiated adenocarcinoma; por2: poorly-differentiated adenocarcinoma, non-solid type; tub: differentiated adenocarcinoma; OS: overall survival; GEM: gemcitabine

	Age	Sex	Localization of PaC	Organization type	Stage	Localization of GC	Organization type	Stage	Other complicated cancers	Chemotherapy	OS
Yasuda, 2006	63	F	Ph	not mentioned	4a(PALN)	Middle third	por2	1A	none	S-1 GEM	12M alive
Kourie, 2013	56	M	Ph	PDAC	4(liver)	Lower third	por	not mentioned	not mentioned	FOLFIRINOX	not mentioned
Kourie, 2013	62	M	Pt	PDAC	4(peritoneal dissemination)	Upper third	not mentioned	not mentioned	not mentioned	FOLFIRINOX	not mentioned
Ohtsubo, 2013	77	M	Ph	PDAC	1B	Middle third	tub	1B	Caecum cancer(Stage2A)	S-1	13M death

The combined patient population (Tables 1, 2) with pancreatic cancer had a median age of 67 years (range: 56-87 years). In ten [6-8,10-12,16-18], two [5,15], and four cases [9,13,14,17], the pancreatic cancer was localized in the pancreatic head, pancreatic body, and pancreatic tail, respectively. Gastric cancer was localized to the fundus, corpus, and antecubital region of the stomach in two [15,17], nine [5-7,10-12,16,18], and five cases [8,9,13,14,17], respectively. The clinical stage of pancreatic cancer was more advanced in most cases. In addition to pancreatic and gastric cancers, there were three cases [5,10,18] of overlapping cancers of other organs such as the bile duct [8], sigmoid colon [10], and caecum [18]. Twelve patients underwent primary tumor resection surgery, and four received chemotherapy alone without surgery.

A two-stage surgical treatment was selected for managing our case. Surgical procedures included pancreaticoduodenectomy (PD) or distal pancreatectomy (DP) plus gastric subtotal resection or greater in cases of stomach fundus and corpus cancer. PD was performed in patients with pancreatic head and gastric antecubital cancers. There was one case of DP plus total gastrectomy performed laparoscopically [15]. Death was confirmed in six of the 12 cases wherein surgery was performed [5,7,9-12]. All procedures were open, with PD or DP and gastric subtotal resection or more. The median postoperative survival of these six patients was 13 months [5,7,9-12].

Three of the four patients who received chemotherapy alone (Table 2) experienced distant metastases [16,17]. The remaining patient had triple cancer, including caecum cancer, and was treated with chemotherapy alone because of the age and invasiveness of surgery [18]. Chemotherapy regimens of choice included gemcitabine and folfirinox as well as S-1 [16-18]. In the case of postoperative chemotherapy, we suggest that the treatment was chosen with more emphasis on the more advanced stage in the surgical specimen [12,14,15]. In the two unresectable cases, folfirinox was chosen as the most aggressive regimen of targeted therapy [17]. 

Ohike et al. (1998) [2], who pathologically examined duplicate cancers, including pancreatic cancer, indicated that the progression of pancreatic cancer was often more extensive than that of duplicate cancers of other organs and the prognosis was largely influenced by the stage of the pancreatic cancer. Eriguchi et al. (2000) [19], who examined 12 cases of multiple cancers including pancreatic cancer, found that the prognosis was determined by pancreatic cancer in many cases. Consistent with these findings, in the 16 cases examined in this study, most patients had more advanced pancreatic cancer than gastric cancer at the time of diagnosis.

Given their anatomic location, it is not expected that the surgical invasiveness of a one-stage resection involving both the pancreas and stomach would be significantly higher than that of pancreatic cancer surgery alone. However, in the 16 cases reviewed in Tables 1, 2, relatively early recurrence and death were observed in cases wherein PD or DP was simultaneously performed with subtotal or more extensive gastric resection. On the other hand, in our case, the patient underwent two-stage resection with chemotherapy in between and survived without recurrence for 49 months after pancreatic cancer surgery and 31 months after gastric cancer surgery.

Shin et al. (2018) [1] reported that the overall survival of patients with overlapping pancreatic and gastric cancer was 33.9 months, which was significantly better compared to those with pancreatic cancer alone (17 months). In the cases reviewed, the overall survival was considerably lower than that. In addition to the small number of cases, the reasons for this might include the fact that the only confirmed deaths were in the cases of laparotomy before 2012; no preoperative chemotherapy was administered; and in few cases, postoperative chemotherapy was administered. Currently, considering that minimally invasive surgeries such as laparoscopic and robot-assisted surgeries have become widespread and multidisciplinary treatment combining chemoradiotherapy has been developed, we suggest that achieving a good treatment course with a one-stage resection is possible. However, in patients who are of an older age group or at high risk for surgical complications due to preexisting disease, similar to the present case, a two-stage treatment along with chemotherapy might be an effective treatment strategy. If the two-stage treatment is chosen, follow-up during the treatment period should be more carefully performed. In the present case, although the patient showed a significant reduction in gastric cancer with chemotherapy after pancreatic cancer surgery, the outcomes necessitated surgical resection. Given that there may have been a period when a less invasive treatment such as endoscopic submucosal dissection (ESD) could have been feasible instead of surgery, closer postoperative upper gastrointestinal endoscopic follow-up should have been considered.

Due to the limited generalizability of findings from a single case study, further research using a prospective design and a larger sample size is necessary to validate and expand these results to other settings.

## Conclusions

We present a case of a two-stage surgical treatment of concurrent and overlapping cancers of the pancreatic head and the upper gastric body. Two-stage surgery might be a viable treatment option in patients with concurrent and overlapping pancreatic cancers, as it can reduce surgical invasiveness and lead to favorable outcomes. The multidisciplinary treatment of gastrointestinal cancers is continually evolving. Moreover, strategies for managing overlapping cancers are likely to expand. Combining chemoradiation therapy with minimally invasive laparoscopic surgery (including robot-assisted surgery) and selecting a treatment plan according to the cancer progression and the patient's general condition are important.

## References

[1] Shin SJ, Park H, Sung YN (2018). Prognosis of pancreatic cancer patients with synchronous or metachronous malignancies from other organs Is better than those with pancreatic cancer only. Cancer Res Treat.

[2] Oike N, Tamura Y, Liu P, Naga Y (1998). Clinicopathological study of multiple cancers including pancreatic cancer. Showa Med Soc J.

[3] (2017). TNM Classification of Malignant Tumours, 8th ed. Wiley-Blackwell.

[4] Arao S, Sadamoto K, Shimazaki K, Higashi N, Baba T, Seo Y, Wakasugi H (1995). A study on cases of multiple cancers including pancreatic cancer and cancer of other organs. Med Care.

[5] Eriguchi N, Aoyagi S, Hara M (2000). A case of synchronous double cancers of the pancreas and stomach. Kurume Med J.

[6] Ishiguro M, Umekita N, Abe H, Inoue A, Kitamura M (2001). A case of advanced gastric cancer and intraductal papillary adenocarcinoma of the pancreas. Jpn J Clin Surg.

[7] Nakatsuji N, Nomi T, Takayama C, Horikawa M, Sugihara S, Maruyama H (2003). A case of synchronous double cancer of pancreatic adenosquamous carcinoma and early gastric cancer. Jpn J Clin Surg.

[8] Mizuno K, Yoshiyama T, Aoki H, Shiozaki S, Ninomiya M, Takakura N (2004). A case of triple cancer of intraductal papillary mucinous adenocarcinoma of the pancreas, bile duct cancer, and gastric cancer in an elderly patient. Jpn J Clin Surg.

[9] Yoshida Y, Inayoshi A, Yagi Y, Arita T (2006). A resected case of pancreatic adenosquamous carcinoma complicated with gastric cancer. Jpn J Clin Surg.

[10] Ouchi A, Isotani M, Kanaoka Y, Maeda A (2009). A case of synchronous triple cancer of the pancreas, gastric, and sigmoid colon in which curative resection was possible. J Jpn Surg Soc.

[11] Tanaka S, Yamamoto T, Ishihara K, Uenishi T, Ohno K (2009). A case of pancreatic adenosquamous carcinoma with gastric cancer that developed rapidly after surgery. J Jap Soc Surg.

[12] Kajiwara M, Morimoto Y, Akamaru Y, Fujii M, Yuba T, Yamazaki Y (2012). Three cases of synchronous double pancreatic cancer treated with pancreaticoduodenectomy. Jpn J Clin Surg.

[13] Santos-Fernández J, Arenal-Vera JJ, Cítores-Pascual MÁ, Fernández-Orcajo P, de-Benito-Sanz M, Benito-Fernández C, Tinoco-Carrasco C (2015). Synchronic gastric and pancreatic ductal adenocarcinoma. A case report. Rev Esp Enferm Dig.

[14] Fang T, Liang TT, Wang YZ, Wu HT, Liu SH, Wang C (2021). Synchronous concomitant pancreatic acinar cell carcin and gastric adenocarcinoma: a case report and review of literature. World J Clin Cases.

[15] Ebihara M, Fujisawa K, Haruta S, Uruga H, Ueno M (2024). Laparoscopic radical total gastrectomy and pancreatosplenectomy for synchronous cancer of the stomach and pancreas. Cureus.

[16] Mikihiko Y, Ken K, Yoshiyuki A (2007). Gemcitabine combined with S-1 chemotherapy is effective for pancreatic and gastric double cancers. Pancreas.

[17] Kourie HR, Markoutsaki N, Roussel H (2013). Double pancreatic and gastric adenocarcinomas: a rare association. Clin Res Hepatol Gastroenterol.

[18] Ohtsubo K, Ishikawa D, Nanjo S (2013). Synchronous triple cancers of the pancreas, stomach, and cecum treated with S-1 followed by pancrelipase treatment of pancreatic exocrine insufficiency. JOP.

[19] Eriguchi N, Aoyagi S, Hara M (2000). Synchronous or metachronous double cancers of the pancreas and other organs: report on 12 cases. Surg Today.

